# Growth in glucose-based medium and exposure to subinhibitory concentrations of imipenem induce biofilm formation in a multidrug-resistant clinical isolate of *Acinetobacter baumannii*

**DOI:** 10.1186/1471-2180-9-270

**Published:** 2009-12-22

**Authors:** Elisabetta Nucleo, Laura Steffanoni, Giulia Fugazza, Roberta Migliavacca, Ernesto Giacobone, Antonella Navarra, Laura Pagani, Paolo Landini

**Affiliations:** 1 Department of Morphological, Eidological and Clinical Sciences, Università di Pavia, Pavia, Italy; 2 Department of Biomolecular Sciences and Biotechnology, Università degli Studi di Milano, Milano, Italy; 3 Microbiological Analysis Laboratory, IRCCS Fondazione San Matteo Hospital, Pavia, Italy; 4 Microbiological Analysis Laboratory IRCCS Fondazione S Maugeri Hospital, Pavia, Italy

## Abstract

**Background:**

*Acinetobacter baumannii *is emerging as an important nosocomial pathogen. Multidrug resistance, as well as ability to withstand environmental stresses, makes eradication of *A. baumannii *difficult, particularly from hospital settings.

**Results:**

Over a six-year period, 73 isolates of *A. baumannii *were collected from infected patients in two hospitals in Italy. While 69 out of the 73 isolates displayed identical multidrug antibiotic resistance pattern, they were susceptible to carbapenems. Genetic profiles of these 69 isolates, determined by Pulsed Field Gel Electrophoresis (PFGE), indicated that they were genetically related and could be clustered in a specific clone, called SMAL. We tested the ability of the SMAL clone to form biofilm, an important determinant for bacterial colonization of the human host and for persistence in the hospital environment. Biofilm formation by *A. baumannii *SMAL, measured as surface adhesion to polystyrene, is strongly affected by growth conditions, being impaired in rich growth media such as LB, while being favoured in glucose-based medium. Surface adhesion in glucose-based media is inhibited by treatment with cellulase, suggesting that it depends on production of cellulose or of a chemically related extracellular polysaccharide. Exposure of *A. baumannii *SMAL to subinhibitory concentrations of imipenem resulted in biofilm stimulation and increased production of iron uptake proteins. Growth in iron-supplemented medium also stimulated surface adhesion, thus suggesting that increased intracellular iron concentrations might act as an environmental signal for biofilm formation in *A. baumannii *SMAL.

**Conclusions:**

Our results indicate that exposure to subinhibitory concentrations of imipenem can stimulate biofilm formation and induce iron uptake in a pathogenic strain of *A. baumannii*, with potential implications on antibiotic susceptibility and ability to persist in the human host.

## Background

Bacteria belonging to the genus *Acinetobacter*, in particular *Acinetobacter baumannii *and the closely related *Acinetobacter *13 TU and gen.sp. 3 (referred to as *Acinetobacter baumannii sensu lato*), are important opportunistic pathogens in hospital-acquired infections (reviewed in [1]).

*A. baumannii *can cause pneumonia, wound infections, urinary tract infections, bacteremia, and meningitis [2,3]. The hospital environment can represent an important reservoir for *A. baumannii *during nosocomial infections; in particular, patients in long-term care facilities can be colonized by *A. baumannii *and carry the bacterium for long periods with no visible symptoms [1]. Ability to persist in the hospital environment is related to multidrug resistance [1,4], which allows *A. baumannii *to survive prolonged antimicrobial therapy in hospitalized patients. Multidrug resistance in *A. baumannii *clinical isolates is mediated by a variety of mechanisms, such as modification of target sites, efflux pumps, enzymatic inactivation of antibiotics, etc. (reviewed in [1]). Carbapenems (*e.g*. imipenem) have been used as antibiotics of choice for treatment of *A. baumannii *infections, but increasing resistance to these antimicrobial agents mediated by β-lactamases of the B and D classes is undermining this option [4-8].

In addition to multidrug resistance, *A. baumannii *is able to withstand environmental stresses such as exposure to detergents [9] and desiccation [10], which can contribute to its persistence in the hospital environment. Biofilm formation is considered an important factor in resistance to stresses and in bacterial colonization and persistence in different environmental niches [11]. It has been reported that ability of *A. baumannii *to form biofilm in laboratory conditions correlates with resistance to complement-mediated bacterial killing [12]. This observation suggests that biofilm formation can contribute to *A. baumannii *survival during host infection, thus representing an important virulence factor. In contrast, studies addressing possible correlation between biofilm and multidrug resistance have produced conflicting results [13-16]. Ability to form biofilm has been reported for numerous *A. baumannii *strains [12-16], and several biofilm determinants, i.e., the *csu *pili [17], and the outer membrane-associated proteins Bap [18] and OmpA [19] have been identified.

In this report, we have characterized *A. baumannii *isolates responsible for nosocomial infections in two hospitals in Italy. We showed that all isolates were genetically related, suggesting that they originate from a single clone, termed SMAL. *A. baumannii *SMAL is not clonally related to known multidrug resistant *A. baumannii *lineages such as European clones I and II [20,21]. We have studied how growth conditions and exposure of *A. baumannii *SMAL to subinhibitory concentrations of imipenem affects its ability to form biofilm, a cellular process with important consequences on sensitivity to antimicrobial agents and on microbial persistence in the human host.

## Results

### Characterization of *Acinetobacter baumannii *clinical isolates

A total of 73 *Acinetobacter baumannii *isolates responsible of various infections were collected from patients in different wards of two Hospitals in Pavia, Italy, between 2002 and 2007. 69 out of 73 isolates showed identical multidrug resistant phenotype, being resistant to fluoroquinolones, aminoglycosides, and most β-lactams; however, they retained susceptibility to carbapenems, tetracycline and to ampicillin/sulbactam (Table 1). The remaining 4 isolates showed different antibiotic susceptibility patterns, including resistance to carbapenems and tetracycline (data not shown). The 69 isolates were characterized by an identical β-lactamase pattern, producing 3 distinct β-lactamases, with pI values of 6.1, 7.0, >8.2, compatible with those of OXA-10, OXA-51-like and AmpC-type enzymes. PCR experiments and direct DNA sequencing using the same primers confirmed the presence of *bla*_OXA-10 _and *bla*_OXA-90 _genes (Table 1). The β-lactamase pattern shown by the isolates is consistent with their susceptibility to carbapenems: indeed, OXA-51-like β-lactamases only possess slow hydrolytic activity against imipenem and result in very little effect on imipenem sensitivity even when overexpressed [22].

**Table 1:** Antimicrobial susceptibility, production of β-lactamases, and pulsotype of the 69 isolates of *A. baumannii *analyzed in this study.

				MIC Method Phoenix (μg/ml)^b^														
							
Number of isolates	Hospital	Sample Source	Ward/unit^a^	TE	A/S	CI	AK	GM	PP	PT	AT	CZ	CP	IP	MP	MIC IP (μg/ml)	β-lactamases	Pulsotype
2	S. Matteo	Blood	Medicine	≤2	8/4	>2	>32	>8	>64	>64/4	>16	>32	>16	≤1	≤1	0.5-1.0	AmpC, OXA-90, OXA-10	SMAL
6	S. Matteo	Sputum	Medicine	≤2	8/4	>2	>32	>8	>64	>64/4	>16	>32	>16	≤1	≤1	0.5-1.0	AmpC, OXA-90, OXA-10	SMAL
2	S. Matteo	Urine	Medicine	4	/	>2	>32	>8	>64	>64/4	>16	>16	/	2	≤1	0.5-1.0	AmpC, OXA-90, OXA-10	SMAL
6	S. Matteo	Soft tissue swab	Medicine	4	8/4	>2	>32	>8	>64	>64/4	>16	>16	>16	≤1	≤1	0.5-1.0	AmpC, OXA-90, OXA-10	SMAL, SMAL 2,
3	S. Matteo	Bronchoaspirate	Medicine	/	8/4	>2	>32	>8	>64	>64/4	>16	>16	>16	≤1	≤1	0.5-1.0	AmpC, OXA-90, OXA-10	SMAL, SMAL 3
3	S. Matteo	Urine	Surgery	4	/	>2	>32	>8	>64	>64/4	>16	>16	/	2	≤1	0.5-1.0	AmpC, OXA-90, OXA-10	SMAL
8	S. Matteo	Wound swab	Surgery	≤2	8/4	>2	>32	>8	>64	>64/4	>16	>16	>16	≤1	≤1	0.5-1.0	AmpC, OXA-90, OXA-10	SMAL
8	S. Matteo	Blood	Surgery	/	/	>2	>32	>8	>64	>64/4	>16	>16	/	2	≤1	0.5-1.0	AmpC, OXA-90, OXA-10	SMAL, SMAL 1
1	S. Matteo	Pus	Surgery	≤2	8/4	>2	>32	>8	>64	>64/4	>16	>16	>16	≤1	≤1	1	AmpC, OXA-90, OXA-10	SMAL
1	S. Matteo	Sputum	Surgery	/	4/2	>2	>32	>8	>64	64/4	>16	>16	>16	≤1	≤1	1	AmpC, OXA-90, OXA-10	SMAL
1	S. Matteo	Soft tissue swab	LTCU	≤2	/	>2	>32	>8	>64	64/4	>16	>16	>16	≤1	≤1	1	AmpC, OXA-90, OXA-10	SMAL
1	S. Matteo	Sputum	LTCU	/	/	>2	>32	>8	>64	64/4	>16	>16	>16	2	≤1	1	AmpC, OXA-90, OXA-10	SMAL
1	S. Matteo	Blood	LTCU	/	/	>2	32	>8	>64	64/4	>16	>16	>16	≤1	≤1	1	AmpC, OXA-90, OXA-10	SMAL
2	S. Matteo	Soft tissue swab	Dermatology	≤2	/	>2	>32	>8	>64	>64/4	>16	>16	>16	≤1	≤1	0.5-1	AmpC, OXA-90, OXA-10	SMAL
1	S. Matteo	Pus	Dermatology	≤2	8/4	>2	>32	>8	>64	>64/4	>16	>16	>16	≤1	≤	1	AmpC, OXA-90, OXA-10	SMAL
2	S. Matteo	Wound swab	Ambulatory	/	/	>2	>32	>8	>64	>64/4	>16	>16	>16	4	2	2	AmpC, OXA-90, OXA-10	SMAL
1	S. Matteo	Urine	Ambulatory	≤2	/	>2	>32	>8	>64	>64/4	>16	>16	/	≤1	≤1	1	AmpC, OXA-90, OXA-10	SMAL
2	S. Matteo	Wound swab	Urology	≤2	/	>2	>32	>8	>64	>64/4	>16	>16	>16	≤1	≤1	0.5	AmpC, OXA-90, OXA-10	SMAL
2	S. Matteo	Urine	Nephrology	/	/	>2	>32	>16	>64	>64/4	>16	>16	/	≤1	≤1	1	AmpC, OXA-90, OXA-10	SMAL
1	S. Matteo	Blood	Haematology	8	/	>2	>32	>8	>64	>64/4	>16	>16	16	≤1	≤1	1	AmpC, OXA-90, OXA-10	SMAL
1	S. Maugeri	Bronchoaspirate	PRU	8	/	>2	>32	>8	>64	>64/4	>16	>16	/	≤1	≤1	1	AmpC, OXA-90, OXA-10	SMAL
7	S. Maugeri	Urine	NRU	/	/	>2	>32	>8	>64	64/4	>16	>16	/	≤1	≤1	1	AmpC, OXA-90, OXA-10	SMAL
2	S. Maugeri	Skin swab	NRU	/	8/4	>2	>32	>8	>64	>64/4	>16	>16	>16	≤1	≤1	0.5	AmpC, OXA-90, OXA-10	SMAL
1	S. Maugeri	Bronchoaspirate	NRU	≤2	/	>2	>32	>8	>64	64/4	>16	>32	/	≤1	≤1	0.5-1	AmpC, OXA-90, OXA-10	SMAL
1	S. Maugeri	Urine	ORU	≤2	8/4	>2	>32	>8	>64	>64/4	>16	>32	/	≤1	≤1	0.5	AmpC, OXA-90, OXA-10	SMAL
1	S. Maugeri	Skin swab	ORU	≤2	8/4	>2	>32	>8	>64	64/4	>16	>32	/	≤1	≤1	0.5	AmpC, OXA-90, OXA-10	SMAL
2	S. Maugeri	Urine	FRU	≤2	8/4	>2	>32	>8	>64	>64/4	>16	>32	>16	≤1	≤1	0.5	AmpC, OXA-90, OXA-10	SMAL

^a ^PRU, pulmonary rehabilitation unit; NRU, neurological rehabilitation unit; ORU, oncological rehabilitation unit. ^b ^TE, tetracicline; A/S, ampicillin/sulbactam; CI, ciprofloxacin; AK, amikacin; GM, gentamicin; PP, piperacillin; PT, piperacillin/tazobactam; AT, aztreonam; CZ, ceftazidime; CP, cefepime; IP, imipenem; MP, meropenem. Ditto marks indicate that the β-lactamase pattern was identical for all the strains tested.

**Figure 1:**
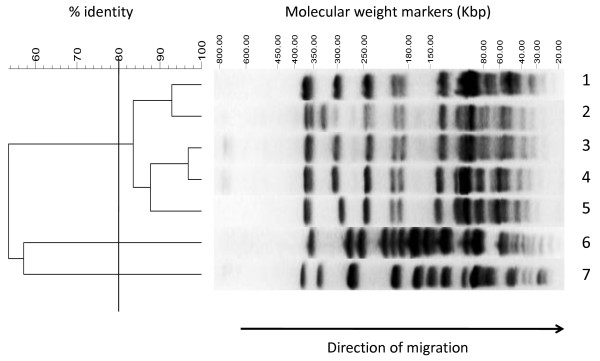
**PFGE profiles of *A. baumannii *genomes after digestion with ApaI restriction nuclease (Lanes 1-7, top to bottom)**. 5 of the 69 isolates identified in this study and analyzed by PFGE are shown (Lanes 1-5). Lane 1, Isolate from urine sample (see Table 1, line 22); Lane 2: Isolate from soft tissue swab (Table 1, line 4); Lane 3: Isolate from blood sample (Table 1, line 8); Lane 4: Isolate from wound swab (Table 1, line 7); Lane 5: Isolate from bronchoaspirate sample (Table 1, Line 5). Isolates were compared to strains representative of European clones I (RUH875, Lane 7) and II (RUH134, Lane 6). Strains belonging to the same clone are clustered at a level of 80% by PFGE with the parameters used as shown by the dendrogram analysis shown on the left.

Genomic DNA was extracted from every *A. baumannii *isolate, digested with ApaI restriction endonuclease, and analysed by PFGE. The dendrogram clearly revealed that all 69 *A. baumannii *isolates showing identical multidrug resistant phenotype displayed more than 80% similarity, with differences in DNA patterns never exceeding 3 DNA restriction fragments. A comparison of a selection of isolates with strains RUH875 and RUH134, representative of European clones I and II, is shown in Figure 1. Our results indicate that, according to the criteria and the cut-off value defined, all isolates belong to the same clone, which was called SMAL, from the hospitals and locations where it had caused outbreaks most frequently (**S**. **M**atteo/**S**. Maugeri Hospitals **A**cute care and **L**ong term care facilities). PFGE experiments indicate that the great majority of isolates belong to a main clonal SMAL subtype, showing 100% genetic similarity, while a smaller number of isolates display a level of genetic relatedness with the SMAL main clonal subtype not lower than 83.5%, defining the clonal subtypes SMAL 1, 2, 3, and 4 (Table 1).

*A. baumannii *strains are notorious for causing recurrent hospital outbreaks, and a few lineages achieve epidemic status, reaching multiple hospitals or communities [23]. Examples include European clones I and II, widespread in continental Europe, and clone III, which is however less relevant in terms of clinical and epidemiological importance [20,21]. The SMAL clone seems to define a novel lineage of *A. baumannii*, as suggested by significant differences in antibiotic resistance pattern (e.g. sensitivity to tetracycline) in comparison to European Clones I and II [20,21]. Lack of genetic relatedness was further confirmed by PFGE experiments, which only showed 57% relatedness between the SMAL clone and isolates belonging to European Clones I and II (Figure 1). Multiplex PCR performed using *ompA*, *csuE*, and *bla*_OXA-51_-like as target genes [24] confirmed these differences (data not shown).

### Biofilm formation by *A. baumannii *clinical isolates

**Figure 2:**
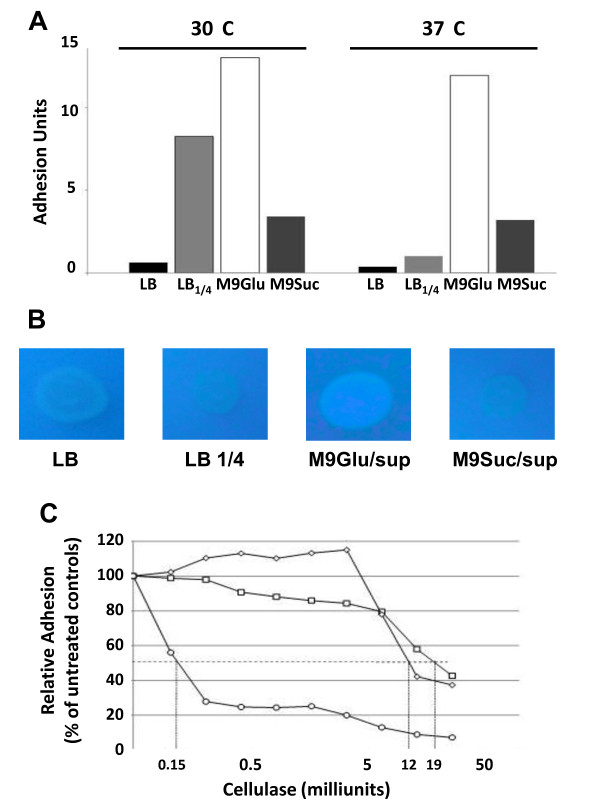
**A. Surface adhesion to polystyrene microtiter plates by *A. baumannii *SMAL clone**. Black bars bacterial cultures grown in LB medium; light grey bars LB1/4 medium; white bars M9Glu/sup; dark grey bars M9Suc/sup. **B. Binding of Calcofluor to *A. baumannii *SMAL clone grown in solid media. C. Inhibition of *A. baumannii *biofilm formation by cellulase treatment: circles, M9Glu/sup medium; diamonds, M9Suc/sup medium; squares, LB1/4 medium**. The horizontal dotted line indicates the 50% inhibition mark. IC_50_'s values are indicated by vertical dotted lines.

The *A. baumannii *isolates belonging to the SMAL clone were tested for their ability to form biofilm, measured as surface adhesion to polystyrene microtiter plates. Biofilm growth is considered an important factor for host colonization [25,26] and for resistance to environmental and cellular stresses [11]. Ability to form biofilm, measured as surface adhesion to polystyrene microtiter plates, was very similar for all *A. baumannii *isolates tested (data not shown); results shown throughout the paper refer to the *A. baumannii *isolate described in Line 22 of Table 1. This isolate was considered representative of the *A. baumannii *SMAL clone since it belongs to the main genotypic subgroup of the SMAL clone (Figure 1) and since it was the first *A. baumannii *to be isolated in this survey. Surface adhesion to microtiter plates by *A. baumannii *SMAL clone was determined in various growth conditions, comparing two growth temperatures (30°C vs. 37°C), and different growth media: the rich peptone-based LB medium, LB medium diluted 1:4 (LB1/4), the M9Glu/sup medium [[27], described in Methods], and the M9Suc/sup in which 0.2% sucrose was added as main carbon source instead of glucose. LB1/4 was tested since it was shown to promote production of adhesion factors in other Gram negative bacteria, such as *Escherichia coli *[28]. We found that biofilm formation by *A. baumannii *SMAL was strongly affected both by growth media and by temperature: indeed, while surface adhesion was very poor in LB medium at either 30°C or 37°C, it was clearly stimulated by growth in LB1/4, although only at 30°C. Finally, growth in M9Glu/sup resulted in efficient surface adhesion both at 30°C and at 37°C, while growth in sucrose-based medium (M9Suc/sup) resulted in much lower levels (Figure 2A). The observation that growth temperature affects biofilm formation in the LB1/4, but not in sugar-based media such as M9Glu/sup, would suggest that this process could be mediated by different mechanisms and by different adhesion factors.

**Figure 3:**
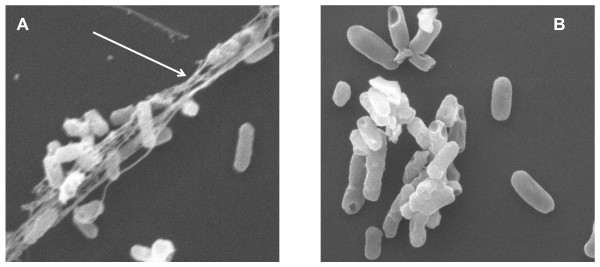
**Transmission electron microscopy images of *A. baumannii *SMAL biofilm-forming cells**. Panel A: *A. baumannii *cells resuspended from biofilm 10,000× magnification. The bundle-like fibers embedding the bacterial cells are indicated by the arrow. Panel B: *A. baumannii *cells resuspended from biofilm and treated with 1 Unit cellulase for 30 minutes, 12,000× magnification.

A major adhesion factor characterized in *A. baumannii *is represented by the *csu *pili described in the *A. baumannii *strain ATCC 19606 [17]. Thus, we measured by Real Time PCR relative expression of the *csuC *and *csuE *genes in different growth media. The *csuC *and *csuE *genes encode respectively a chaperone involved in pili assembly and the pilus major subunit. Expression of *csu *genes was hardly detectable in all growth conditions (data not shown). Consistent with this result, we could not detect any production of *csu *pili in *A. baumannii *SMAL by electron microscopy, regardless of growth conditions (Figure 3 and data not shown). This result would suggest that production of *csu *pili, and thus their contribution to surface adhesion, might be limited in this strain. In addition to *csu *pili, *A. baumannii *19606 biofilm is characterized by efficient binding to Calcofluor [17], a fluorescent dye which binds specifically to cellulose and chitin; this observation suggests that cellulose, which is produced as an extracellular polysaccharide (EPS) in many bacteria [29-32], might be a biofilm determinant in *A. baumannii*. To detect possible production of cellulose, we grew *A. baumannii *SMAL on different solid media supplemented with Calcofluor. Interestingly, Calcofluor binding was detected on M9Glu/sup solid medium, but not on M9Suc/sup or in either peptone-based media (LB or LB1/4), suggesting that growth on glucose induces production of Calcofluor-binding EPS in *A. baumannii *SMAL (Figure 2B). In order to test the possible role of this EPS as an adhesion factor, we tested surface adhesion to polystyrene in different growth media in the presence of the cellulose-degrading enzyme cellulase (Figure 2C). Surface adhesion was efficiently inhibited by low amounts of cellulase when *A. baumannii *SMAL was grown in M9Glu/sup (50% inhibition at 0.15 Units cellulase, Figure 2C), thus suggesting that surface adhesion is mediated by cellulose production. In contrast, cellulase was only able to impair surface adhesion at much higher concentrations when *A. baumannii *SMAL was grown either in M9Suc/sup or in LB1/4 media (50% inhibition at ca. 12 and 19 Units cellulase, respectively, Figure 2C). At these amounts of cellulase, inhibitory effects are likely due to non-specific effects such as changes in surface tension or other physico-chemical properties of the medium. Cellulase effects in LB medium were not tested due to the very inefficient biofilm formation in this medium (Figure 2A). To further verify the possible role of cellulose-related EPS as an adhesion factor, *A. baumannii *SMAL biofilm formed on microtiter plates by cells growing in M9Glu/sup medium was resuspended in 50 mM phosphate buffer pH 6.0 by vigorous pipetting and incubated 30 minutes either in the presence or in the absence of 1 U cellulase prior to fixation with gluteraldehyde and visualization by transmission electron microscopy. Figure 3 shows that *A. baumannii *SMAL cells recovered from the biofilm appear embedded in bundle-like filaments (Panel 3A), which disappear upon cellulase treatment (Panel 3B), further confirming direct involvement of cellulose in cell-cell aggregation.

In addition to its role of adhesion factor, cellulose, as well as other EPS, can protect bacterial cells from environmental stresses such as desiccation and oxidative stress [11,29]. Thus, we tested the *A. baumannii *SMAL clone grown either in M9Glu/sup or in LB1/4 for resistance to desiccation and to challenge with H_2_O_2_. *A. baumannii *SMAL displayed high levels of resistance to both stresses, which was expected since this is a common feature for the *Acinetobacter *genus [1]; growth in different media did not significantly affect its resistance level (data not shown), suggesting that, in *A. baumannii *SMAL, cellulose production might be more related to surface adhesion than to resistance to environmental stresses.

### Exposure to subinhibitory concentrations of imipenem affects biofilm formation

**Figure 4:**
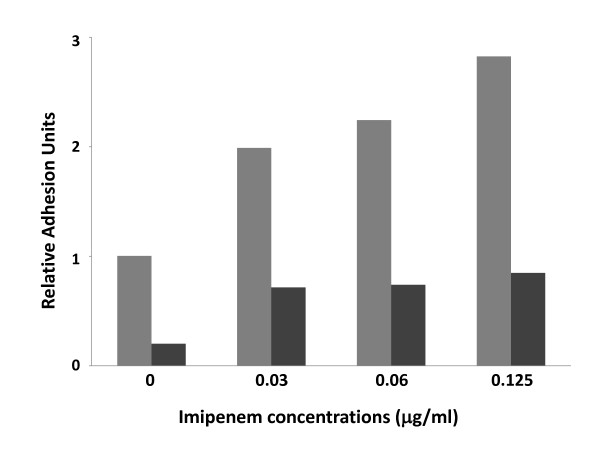
**Surface adhesion by *A. baumannii *SMAL clone grown in M9Glu/sup medium at 30°C in the presence of subinhibitory imipenem concentrations**. Grey bars: untreated samples; black bars: samples treated with 1 Unit cellulase.

The *A. baumannii *SMAL clone is sensitive to carbapenems such as imipenem (Table 1). However, in many cases, imipenem treatments failed to eradicate the *A. baumannii *SMAL clone from patients, often resulting in relapses. We investigated the possibility that, although sensitive to imipenem in standard Minimal Inhibitory Concentration (MIC) determination assays, the *A. baumannii *SMAL clone might possess mechanisms of resistance or tolerance to this antibiotic. Exposure to subinhibitory concentrations of antibiotics can result in the induction of adaptive responses and in biofilm stimulation [33], which appears to increase tolerance to antibiotics via different molecular mechanisms (reviewed in [34]). Thus, we tested the effect of subinhibitory concentrations of imipenem on biofilm formation by *A. baumannii *SMAL: concentrations of imipenem ranging between 0.03 and 0.125 μg/ml, which correspond respectively to 1/16 and 1/4 of the MIC of imipenem in M9Glu/sup medium, resulted in biofilm stimulation by up to 3-fold, both at 30°C (Figure 4) and at 37°C (data not shown). Growth rate was not impaired by imipenem at any of the concentrations tested. In contrast, treatment of *A. baumannii *SMAL with subinhibitory concentrations of tetracycline did not result in any significant induction of biofilm formation (data not shown), suggesting that biofilm induction is a specific effect of imipenem. Since in M9Glu/sup medium surface adhesion by *A. baumannii *SMAL is mediated by cellulose production (Figure 2C), we tested whether imipenem-induced biofilm stimulation could be inhibited by treatment with cellulase. As shown in Figure 3, although cellulase did affect biofilm formation both in the presence and in the absence of imipenem, the extent of biofilm stimulation induced by the antibiotic is very similar (ca. 3-fold) regardless of the presence of cellulase. This observation seems to suggest that exposure to subinhibitory imipenem concentrations induce production of a cellulase-resistant adhesion factor. To understand if imipenem-dependent biofilm stimulation is specific for *A. baumannii *SMAL, we tested the effects of subinhibitory imipenem concentration on biofilm formation in *A. baumannii *strains RUH875 and RUH134, representative of European clones I and II. In the absence of imipenem, both strains could form biofilm to a similar extent as *A. baumannii *SMAL (data not shown). MICs of imipenem for RUH875 and RUH134 in M9Glu/sup medium were 0.5 and 0.25 μg/ml, again very similar to the MIC for *A. baumannii *SMAL. Unlike *A. baumannii *SMAL, however, exposure to subinhibitory concentrations of the antibiotic failed to stimulate surface adhesion in these strains (data not shown).

**Figure 5:**
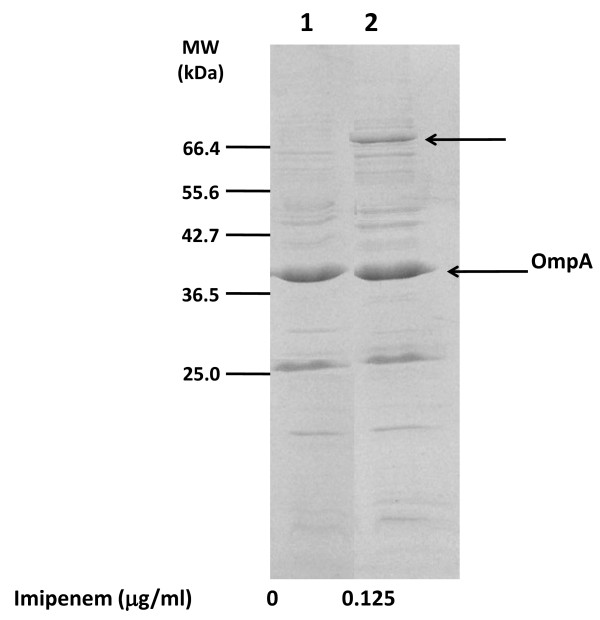
**SDS-PAGE of membrane fractions of *A. baumanni *SMAL clone**: the arrows point to the 70 KDa bands showing different levels of expression in cultures treated with imipenem. The band at ca. 40 KDa was identified by MALDI-TOF as OmpA the major outer membrane protein in *A. baumannii*. Molecular Weight standards are shown.

In order to identify possible imipenem-dependent biofilm determinants we compared the patterns of membrane-associated proteins of *A. baumannii *SMAL grown either in the absence or in the presence of 0.125 μg/ml imipenem (1/4 MIC). Exposure to subinhibitory imipenem concentrations clearly affected the intensity of a protein band with the apparent molecular weight of ca. 70 KDa (Figure 5). The 70 KDa bands from both the control and the imipenem-treated samples were excised from the gel, and the proteins were digested with trypsin and identified through MALDI-TOF analysis as previously described [35]. The 70 KDa bands were identified as a mixture of three polypeptides, all involved in metal uptake: the OprC protein, a copper receptor, was found both in control and imipenem-exposed bacterial cultures. In contrast, two proteins involved in iron uptake, a ferrichrome receptor protein and a TonB-dependent siderophore, were only found in the membrane of imipenem-exposed cultures (Table 2). We tested the possibility that increased production of iron uptake proteins upon exposure to subinhibitory imipenem concentrations could be due to transcription activation of the corresponding genes. Relative transcription levels of the ferrichrome receptor protein- and the TonB-dependent siderophore-encoding genes were determined by Real Time PCR experiments, which showed that transcription of both genes is activated by 0.125 μg/ml imipenem (1/4 the MIC) by 3.5-fold (Table 2).

**Table 2:** Identification of membrane proteins induced by exposure to subinhibitory imipenem concentrations.

Protein identified by MALDI-TOF and/or corresponding gene in *A. baumannii *ATCC 17978	Expected Molecular Weight (KDa)	Protein function	Conditions in which proteins are produced	Gene expression in the presence of imipenem (fold induction)
OprC (A1S_0170)	67,700	Putative outer membrane copper receptor	Both in control and in imipenem-induced cultures	N.D.

(A1S_1921)	71,742	Ferrichrome-iron receptor	Imipenem-induced cultures	3.51

(A1S_1063)	73,034	TonB-dependent siderophore receptor	Imipenem-induced cultures	3.39

Genes encoding the identified proteins are identified with the annotation number for *A. baumanni *ATCC 17978 strain [52].

### Effects of iron on biofilm formation

**Figure 6:**
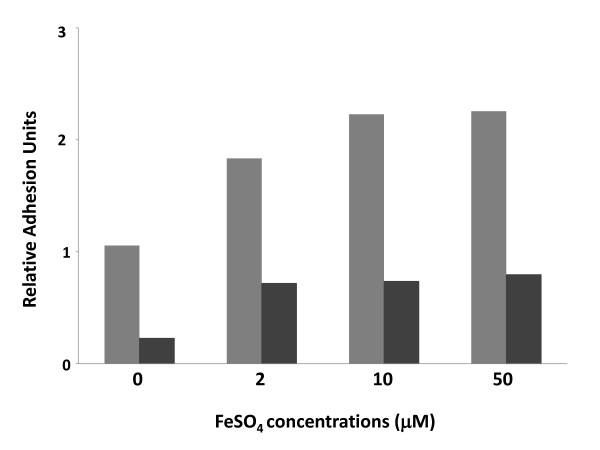
**Surface adhesion by *A. baumannii *SMAL clone grown in M9Glu/sup medium at 30°C in the presence of FeSO_4_**. Grey bars: untreated samples; black bars: samples treated with 1 Unit cellulase.

Our results indicate that subinhibitory imipenem concentrations positively affect both surface adhesion (Figure 4) and iron uptake (Figure 5, Table 2). In most bacteria, iron is an important environmental signal for production of adhesion factors and biofilm formation [36,37]. Thus, it is possible that biofilm stimulation by imipenem might depend upon higher intracellular iron concentration mediated by increased production of iron uptake proteins. To verify this hypothesis, we tested the effects of iron on surface adhesion by *A. baumannii *SMAL. Addition to the M9Glu/sup medium of FeSO_4 _at concentrations ranging between 2 and 50 μM led to a 2.5-fold stimulation of surface adhesion (Figure 6). Similar to what observed for subinhibitory imipenem concentrations, iron-dependent biofilm stimulation takes place even in the presence of cellulase, thus suggesting that it is not mediated by increased production of cellulose (Figure 6). We tested the possibility that biofilm stimulation either by iron or by subinhibitory imipenem concentrations could be mediated by increased expression of the pili-encoding *csu *genes. However, Real Time PCR experiments showed no significant changes either in *csuC *or *csuE *transcription in response to exposure either to 0.125 μg/ml imipenem or to 50 μM FeSO_4 _(data not shown).

## Discussion

In this work, we have reported the isolation and characterization of an *A. baumannii *strain responsible for outbreaks both in Acute Care and in Long-Term Care Facilities in two Italian hospitals. *A. baumannii *isolates showed a distinct antibiotic resistance pattern, being resistant to most aminoglycosides and β-lactams, but sensitive to carbapenems and tetracycline (Table 1). Analysis of the isolates by PFGE suggests that they belong to a single lineage, unrelated to *A. baumannii *European clones I and II (Figure 1). This *A. baumannii *clone, named SMAL (acronym of the hospitals and wards where it was isolated), was repeatedly isolated over a six-year period, and was able to cause different kinds of infections, ranging from urinary tract infections to bacteremia (Table 1), thus showing significant potential as pathogenic strain as well as ability to persist in the hospital environment.

Ability to form biofilm plays an important role both in survival within the host and in persistence of *A. baumannii *in hospital environments, thus leading to recurrent nosocomial infections [1]. Our results show that biofilm formation by the *A. baumannii *SMAL clone, measured as ability to adhere to polystyrene microtiter plates, is strongly affected by growth conditions, being inhibited in the rich, peptone-based, LB medium (Figure 2A). 1:4 dilution of the LB medium was enough to stimulate surface adhesion, which, however, was further increased by growth in glucose-based medium (Figure 2A). Biofilm stimulation by growth on glucose was also observed for strains RUH875 and RUH134, representative of epidemic European clones I and II (data not shown), in line with similar effects reported for the *A. baumannii *strain ATCC 19606 [17]. These observations strongly suggest that, to fully evaluate biofilm proficiency of *A. baumannii *clinical isolates, biofilm assays should be carried out, not only in peptone-based media, as reported in various studies [12-14], but also in glucose-based media. Binding to the fluorescent dye Calcofluor (Figure 2B) and biofilm sensitivity to cellulase (Figure 2C) strongly suggest that growth on glucose-based medium triggers production of cellulose, or possibly of an EPS containing a β-1,4-glucan portion. Initial attempts to identify the chemical nature of the EPS produced by *A. baumannii *SMAL would indeed suggest that its composition is very complex (data not shown). Production of a Calcofluor-binding EPS was not stimulated by sugars other than glucose, such as sucrose (Figure 2B), as well as lactose and arabinose (data not shown), thus suggesting that glucose is a specific inducer of EPS production. Identification of a β-1,4-glucan-containing EPS as an adhesion factor, and of its dependence on glucose, is relevant for the understanding of which biofilm determinants are produced by *A. baumannii *in different environments and in different body sites during host colonization. Indeed, glucose concentration in blood, but not in other *A. baumannii *infection sites such as in the urinary tract, are similar to the concentrations used in our experiments and would thus be able to induce EPS production. In addition to promoting cell adhesion, production of cellulose might contribute to protection from macrophage killing, a role proposed for other bacterial EPS such as alginate in *P. aeruginosa *[38]. We have identified putative glycosyltransferase-encoding genes in the *A. baumannii *SMAL genome that might be involved in EPS biosynthesis. However, attempts to inactivate genes possibly involved in EPS biosynthesis and to assess their role have not been successful so far.

Although *A. baumannii *SMAL clone is sensitive to imipenem *in vitro *(Table 1), treatments with this antibiotic often failed to clear the patients from infections (data not shown), thus suggesting that *A. baumannii *SMAL might show tolerance to imipenem during host infection. We investigated the possibility that *A. baumannii *SMAL sensitivity to imipenem might be affected by different growth conditions and/or by biofilm formation. MIC for imipenem in glucose-based medium was lower compared to the MIC in peptone-based growth media (0.5 vs. 2 μg/ml; data not shown), thus suggesting that biofilm formation in M9Glu/sup does not result in increased resistance to imipenem. However, exposure to subinhibitory imipenem concentrations (0.03-0.125 μg/ml) results in a 3-fold stimulation of surface adhesion (Figure 4). Interestingly, imipenem-dependent biofilm stimulation appears to be distinctive for *A. baumannii *SMAL, since it was not observed in strains RUH875 and RUH134, representative of epidemic European clones I and II. It is likely that this specific response to imipenem might contribute to *A. baumannii *SMAL pathogenic and epidemic potential.

In addition, exposure to subinhibitory imipenem concentrations increased production of ferrichrome receptor protein and of TonB-like siderophore receptor protein, both involved in iron uptake (Figure 5, Table 2). Imipenem-dependent increase in expression of iron uptake proteins is probably part of a more general response to a cellular stress, rather than being induced by an actual reduction in available iron by imipenem at the concentrations tested. Iron uptake proteins play a key role during host infection by various bacteria [39]; consistent with this function, pathogenic *A. baumannii *strains possess a large number of iron uptake genes in comparison to environmental isolates [40]. Induction of ferrichrome receptor and the TonB-like siderophore receptor proteins by imipenem appears to take place via transcription activation of the corresponding genes (Table 2). Thus, exposure to subinhibitory imipenem concentrations can trigger the production of both biofilm determinants and iron uptake proteins, in what appears to be a co-ordinated response to cellular stresses. Direct connection between iron uptake and biofilm formation is also suggested by the observation that increased FeSO_4 _concentrations in the growth medium can act as a positive environmental signal for surface adhesion in the *A. baumannii *SMAL clone (Figure 6). Our results suggest that neither cellulose nor *csu *pili are responsible for iron-dependent increase in surface adhesion: interestingly, a recent report shows that adherence to human airway epithelial cell is independent of *csu *pili [41], thus suggesting that important adhesion and virulence factors of *A. baumannii *are yet to be identified.

## Conclusions

In the present study we have characterized a novel multidrug-resistant, pathogenic strain of *A. baumannii *(*A. baumannii *SMAL clone). We have highlighted the importance of environmental signals such as glucose and iron availability for biofilm formation by this strain. Our results suggest that exposure to subinhibitory imipenem concentrations stimulates iron uptake, which in turn leads to an increased production of adhesion factors and biofilm formation. Induction of biofilm formation by subinhibitory antibiotic concentration, even when it does not directly result in increased antibiotic resistance *in vitro*, can nonetheless protect bacteria against killing by antimicrobials during host infection [33,42]. Understanding of the molecular mechanism of imipenem-induced biofilm formation could provide useful information for the design of more effective protocols in antimicrobial therapy.

## Methods

### Bacterial identification

A total of 69 *A. baumannii *non-replicated isolates, recovered between 2002 and 2007 from patients in medical, surgical and long-term care wards, were included in the study. Isolates were collected in two different hospitals in Pavia, Italy: the "*I.R.C.C.S. Fondazione S. Maugeri*", a Long-Term Care Facility, and the "*I.R.C.C.S. Fondazione S. Matteo*", an Acute Care Hospital. The isolates were initially identified using the automatic systems Vitek 2 (BioMérieux, Marcy-l'Etoile, France) and Phoenix (Becton Dickinson, Sparks, MD). Detection of *bla*_OXA-51_-like alleles by PCR was used to confirm the identification of the isolates as *A. baumannii *[43]. Antibiotic susceptibility was determined using Phoenix System, Panel NMIC/ID4 (Becton Dickinson Diagnostic Systems). Carbapenems susceptibility was confirmed by broth macrodilution procedures according to CLSI guidelines (CLSI document M100-S18). *Escherichia coli *ATCC 25922 and *Pseudomonas aeruginosa *ATCC 27853 were used as reference quality control strains of *in vitro *susceptibility tests. An isolate was defined as multidrug resistant if resistant to at least three classes of antibiotics commonly used in the treatment of *A. baumannii *infections.

### Characterization of β-lactamases

Analytical isoelectric focusing (IEF) of crude extracts, visualization of β-lactamase bands by nitrocefin, and detection of their activity by a substrate overlaying procedure were performed as described [44]. Known producers of various β-lactamases (TEM-1, TEM-2, TEM-7, TEM-8, TEM-9, TEM-12, SHV-1, SHV-2 and SHV-5) were used as controls. PCR amplification of *bla*_OXA-51 _and of *bla*_OXA-10_-like alleles was carried out with primers OXA-51-F (5'-CTCTTACTTATMACAAGCGC-3') and OXA-51-R (5'-CGAACAGAGCTAGRTATTC-3') (for *bla*_OXA-51_) and with primers OXA-10-F (5'-GTCTTTCGAGTACGGCATTA-3') and OXA-10-R (5'-ATTTTCTTAGCGGCAACTTAC-3') for *bla*_OXA-10_-like [45]. The PCR amplicons of *bla*_OXA-51 _and *bla*_OXA-10 _genes were purified using the kit Quantum Prep PCR Kleen Spin Columns (BioRad) and subjected to direct sequencing. PCR products were sequenced on both strands with an Applied Biosystems sequencer. The nucleotide sequences were analysed with the BLAST program.

### Genotyping of *A. baumannii *isolates

Genetic relatedness among *A. baumanni *isolates was investigated by pulsed-field gel electrophoresis (PFGE): genomic DNA was digested with ApaI restriction enzyme and restricted fragments were separated on a CHEF-DR II apparatus (Bio-Rad) for 20 h at 14°C. Bacteriophage λ concatemers were used as DNA size markers. DNA restriction patterns of scanned gel pictures were interpreted following cluster analysis with Fingerprinting II version 3.0 software (Bio-Rad) using the unweighted pair-group method with arithmetic averages (UPGMA). The Dice correlation coefficient was used with a 1.2% position tolerance to analyse the similarities of the banding patterns. Only bands larger than 48 Kb were considered for the analysis. Isolates showing more than three DNA fragment differences and a similarity of <80% were considered to represent different PFGE types, while isolates with less than three fragment differences and a similarity of >80% were considered as belonging to the same PFGE subtype, following the criteria for genetic characterization using PFGE described in the literature [23,46,47]. *A. baumannii *RUH875 and RUH134 were used as reference strains representative of the European clonal lineages I and II, respectively [20,48].

### Biofilm formation assays and determination of EPS production

Biofilm formation in microtiter plates was determined as described [49]. Bacterial cells were grown overnight in microtiter plates (0.2 ml) either at 30°C or 37°C. Bacterial growth in the liquid culture was determined by optical density at 600 nm (OD_600 nm_) and the liquid culture was removed. Microtiter plates were washed with 0.1 M phosphate buffer (pH 7.0), and the biofilm cells attached to the microtiter plate wells were stained for 20 min with 1% crystal violet (CV) in ethanol, washed, and dried. Crystal violet staining was visually assessed and the microtiter plates were scanned. For semi-quantitative determination of biofilms, CV-stained cells were resuspended in either 0.2 ml of 70% ethanol. The absorbance at 600 nm (Abs_600 nm_) of the resuspended CV was determined and normalized to the OD_600 nm _of the corresponding grown cell density: this value corresponds to the "adhesion units". To test biofilm sensitivity to cellulase, bacterial cultures were grown in the presence of cellulase from *Trichoderma reesei *ATCC 26921 (5 mg/ml, 700 U/ml, Sigma). For detection of cellulose production by binding to the fluorescent dye Calcofluor (CF), bacteria were grown overnight in a microtiter plate, and the cultures were spotted, using a replicator, on solid media to which 0.005% Calcofluor (for CF medium) was added after autoclaving. Bacteria were grown for 18-20 h at 30°C; staining was better detected after 24-48 h of additional incubation at 4°C.

### SDS-PAGE analysis of membrane proteins

*A. baumannii *cultures (100 ml) were grown in defined M9 medium, supplemented with 0.02% peptone and 0.01% yeast extract, to which 0.2% glucose was added as main carbon source (M9Glu/sup, [27]). Cultures were grown at 30°C up to 0.1 OD_600 nm _prior to addition of 0.125 μg/ml imipenem (1/4 the MIC). Both control and treated cultures were harvested 3 hours after imipenem addition, at an OD_600 nm _>1.0. Bacterial cells were harvested by centrifugation at 4,000 × g for 10 min at 4°C and washed with 5 ml 0.1 M phosphate buffer pH 7.0 (PB). The pellet was resuspended in 2 ml PB with addition of 100 μg/ml lysozyme and 1 mM EDTA pH 8.0 and incubated at room temperature for 10 minutes. Cells were disintegrated using a French Press and centrifuged as above to remove unbroken cells. The low-speed centrifugation supernatant was then centrifuged at 30,000 × g for 30 minutes at 4°C to separate the cytoplasm (supernatant) and the membrane fraction (pellet). The pellet was resuspended in 1 ml of PB. Protein concentrations were determined and 25 μg of total proteins was loaded onto a 10% sodium dodecyl sulfate-polyacrylamide gel (SDS-PAGE). Bands of interest were excised from the gel and the corresponding proteins were identified by matrix-assisted laser desorption ionization-time of flight (MALDI-TOF) analysis of the peptide generated by in-gel trypsin digestion ([35]; performed by CEINGE, University of Naples, Italy http://www.ceinge.unina.it/).

### Measurement of gene expression by Real Time-PCR

Gene expression determination was performed using Real Time-PCR as previously described [29]. RNA was extracted from bacterial cultures grown as for membrane protein extraction. Production of cDNAs was obtained by reverse transcription using 1 μg total RNA, along with negative control samples incubated without reverse transcriptase. Primer sequences for genes of interest were designed based on the available genome sequences for *A. baumannii *and were tested in PCR experiments on *A. baumannii *SMAL genomic DNA to verify the presence of the gene and the correctness of the expected products. Primer sequences were as follows: fchR_for: 5'-ACGTCAAGCGGTTGCTCCAT-3', fchR_rev: 5'-CCTGTAATCGGGTCTGTTGG-3', tonB_for: 5'-ATGGCAAGATACCGATGCCC-3', tonB_rev: 5'-CCGATATCTTCGCTTGAGCG-3', csuC_for: 5'-GCCCGCCTGTAGCCAAAATT-3', csuC_rev: 5'-GAAGCATCTTGCTCGTTGCC-3', csuE_for: 5'-TAGCGGGCCTGATGGCAATT-3', csuE_rev: 5'-ACCCAGGGCTCTCAAAGAAG-3', 16S_for: 5'-TGTCGTCAGCTCGTGTCGTGA-3', 16S_rev: 5'-TGATGACTTGACGTCGTCCCC-3'. Each Real Time PCR experiment was performed in triplicate and included negative control samples, which never showed significant threshold cycles. The relative transcript amounts were determined using 16S rRNA as the reference gene ([Ct_Gene of interest_-Ct_16S_] = ΔCt value). The results are the average of at least three independent experiments showing standard deviations ≤10%.

### Other methods

Resistance to desiccation was performed as described in [29]. Sensitivity to oxidative stress was determined by treatment with hydrogen peroxide (H_2_O_2_), as described previously [50]. Transmission electron microscopy analysis was performed as described [51].

## Authors' contributions

EN and RM performed the genetic characterization of the isolates. LS and GF carried out experimental work on adhesion factors. EG and AN performed the initial isolation of *A. baumannii*. LP supervised the genetic characterization of the isolates. PL supervised the experiments related to the identification of the adhesion factors and wrote the manuscript, which was revised and approved by all authors.
